# Development of Diagnosis Model for Early Lung Nodules Based on a Seven Autoantibodies Panel and Imaging Features

**DOI:** 10.3389/fonc.2022.883543

**Published:** 2022-04-21

**Authors:** Leidi Xu, Ning Chang, Tingyi Yang, Yuxiang Lang, Yong Zhang, Yinggang Che, Hangtian Xi, Weiqi Zhang, Qingtao Song, Ying Zhou, Xuemin Yang, Juanli Yang, Shuoyao Qu, Jian Zhang

**Affiliations:** ^1^ Department of Pulmonary Medicine, Xijing Hospital, Air Force Medical University, Xi’an, China; ^2^ National Science Library, Chinese Academy of Sciences, Beijing, China; ^3^ Department of Radiology, Xijing Hospital, Air Force Medical University, Xi'an, China; ^4^ Amoy Diagnostics Co. Ltd , Xiamen, China

**Keywords:** autoantibodies, neural network, early diagnosis, lung cancer, radiology

## Abstract

**Background:**

There is increasing incidence of pulmonary nodules due to the promotion and popularization of low-dose computed tomography (LDCT) screening for potential populations with suspected lung cancer. However, a high rate of false-positive and concern of radiation-related cancer risk of repeated CT scanning remains a major obstacle to its wide application. Here, we aimed to investigate the clinical value of a non-invasive and simple test, named the seven autoantibodies (7-AABs) assay (P53, PGP9.5, SOX2, GAGE7, GUB4-5, MAGEA1, and CAGE), in distinguishing malignant pulmonary diseases from benign ones in routine clinical practice, and construct a neural network diagnostic model with the development of machine learning methods.

**Method:**

A total of 933 patients with lung diseases and 744 with lung nodules were identified. The serum levels of the 7-AABs were tested by an enzyme-linked Immunosorbent assay (ELISA). The primary goal was to assess the sensitivity and specificity of the 7-AABs panel in the detection of lung cancer. ROC curves were used to estimate the diagnosis potential of the 7-AABs in different groups. Next, we constructed a machine learning model based on the 7-AABs and imaging features to evaluate the diagnostic efficacy in lung nodules.

**Results:**

The serum levels of all 7-AABs in the malignant lung diseases group were significantly higher than that in the benign group. The sensitivity and specificity of the 7-AABs panel test were 60.7% and 81.5% in the whole group, and 59.7% and 81.1% in cases with early lung nodules. Comparing to the 7-AABs panel test alone, the neural network model improved the AUC from 0.748 to 0.96 in patients with pulmonary nodules.

**Conclusion:**

The 7-AABs panel may be a promising method for early detection of lung cancer, and we constructed a new diagnostic model with better efficiency to distinguish malignant lung nodules from benign nodules which could be used in clinical practice.

## Introduction

Lung cancer is the most common malignant tumor as well as the leading cause of cancer-related deaths worldwide. According to the newest global statistics, approximately 85% of lung cancers are identified at advanced stages that are incurable (1, 2). This is mainly ascribed to the ineffective and insufficient methods for early diagnosis. As is known, 5-year survival could reach 77%-92% in patients with stage I lung cancer while it is less than 10% in stage IV (3, 4). Therefore, there is an urgent need to develop robust methods to detect lung cancer at the early stage in order to improve the long-term survival.

To date, radiographic screening is the major approach for early detection of lung cancer. The National Lung Screening Trial (NLST) reported that low-dose computed tomography (LDCT) can reduce lung cancer mortality by 20% (5). The NELSON study suggested that LDCT could detect more and smaller cancers especially in the early stage (6). However, there are several problems including low sensitivity and a high false-positive rate which result in excessive diagnosis and treatment, and repeated LDCT scanning that could increase the risk of radiation-related cancers and psychological stress like anxiety (7).

With the deep understanding of immunoediting theory, previous studies have suggested that the immune system could recognize over-expressed, mutated, misfolded, or aberrantly degraded self-proteins from tumor cells in the early stage of carcinogenesis (8). Abnormal proteins would acquire immunogenicity and lead to the formation of autoantibodies (AABs) *via* humoral immune responses (9). Furthermore, the signal amplification effect of the immune system could cause some AABs to be captured several months or years earlier than the appearance of symptomatic cancer (10). Compared to other traditional tumor markers mainly for efficient monitoring rather than early diagnosis, AABs possess unique advantages for early detection including preclinical expression, high specificity, and long-term stability. Considering the relatively low sensitivity of a single AAB, a reasonable combination of AABs could improve sensitivity and diagnostic yield. In 2008, a panel of AABs (P53, C-myc, HER2, NYESO-1, GAGE, MUG1, and GBU4-5) showed a sensitivity of 5%-36% and specificity of 96%-100% *via* an individual antibody (11). However, when using these AABs as a panel, the sensitivity increased up to 76%. Moreover, a recent study suggested that addition of AABs into a new panel (P53, NYESO-1, GAGE, GBU4-5, SOX2, MAGE4, and HuD) could further improve the sensitivity and specificity (12). Now EarlyCDT-Lung has been widely applied in clinical practice overseas (13). More recently, Ren et al. performed a prospective study to investigate the clinical value of a 7-AABs (P53, PGP9.5, CAGE, GBU4-5, SOX2, MAGE7, and MAGEA1) panel in the early detection of lung cancer in a Chinese population (14). They reported this panel had high specificity but relatively low sensitivity in patients presenting with ground-glass nodules (GGNs) and/or solid nodules. Therefore, we would like to construct a new diagnostic model combining different types of data including autoantibodies panels, LDCT, and clinical characteristics of patients to predict early lung nodules.

Over the decades, machine learning has been considered an important method showing remarkable performance on most clinical prediction tasks compared with traditional methods (15). The neural network model could integrate both continuous and discrete data of patients, which shows great advantages over logistic regression (16), and based on the 7-AABs, LDCT data, and clinical characteristics of patients we collected, a new neural network diagnostic model was proposed especially for early stage lung cancer, showcasing a favorable result both in the test set and validation set.

In this study, we summarize and analyze the features of a 7-AABs panel in detection of lung cancer and lung nodules in routine clinical practice in our center. And we further propose a comprehensive diagnostic model including clinical and imaging features in combination with the 7-AABs of patients predicting benign or malignant properties in early lung nodules.

## Method and Materials

### Samples Collection

**Figure 1:**
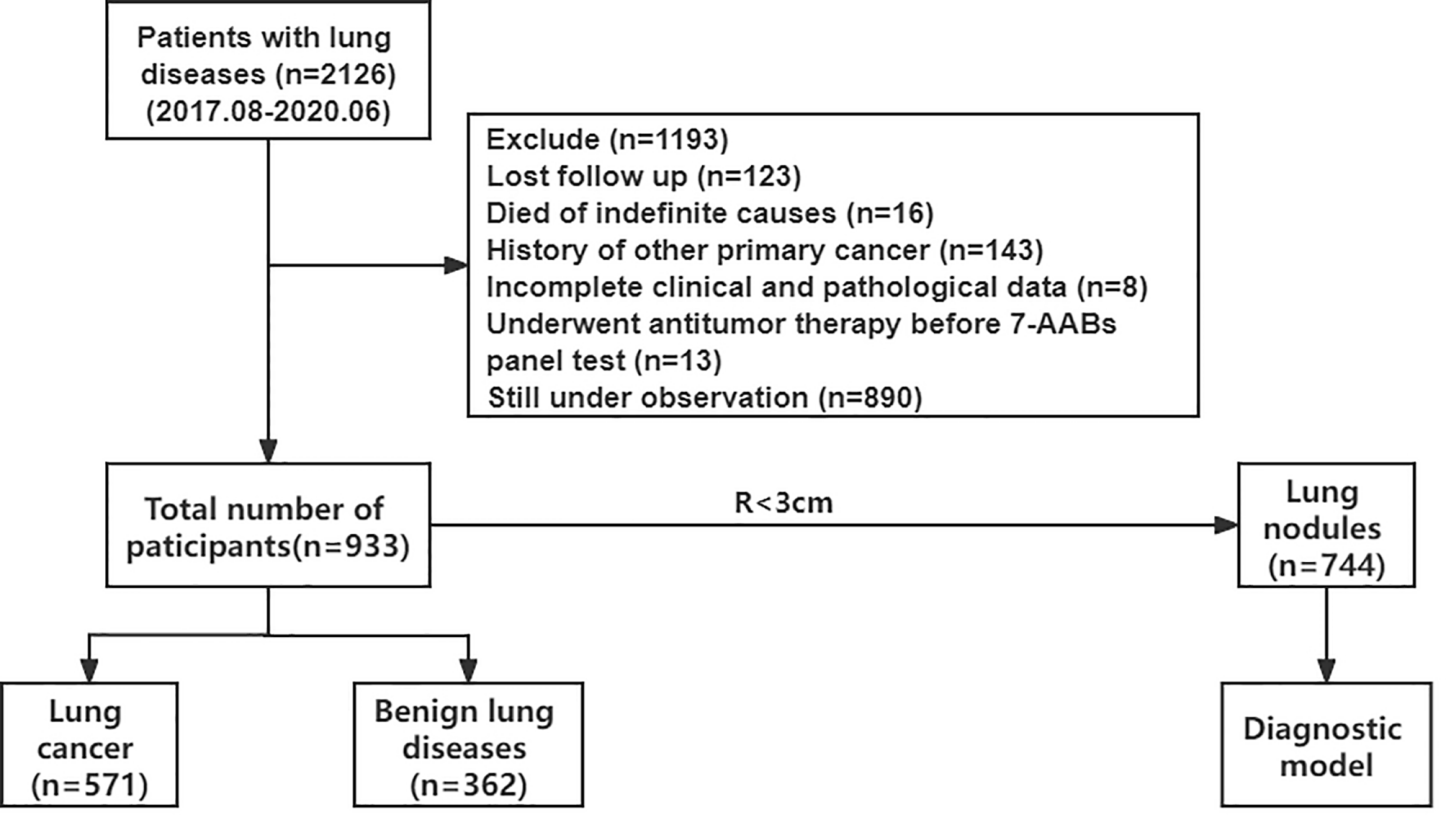
Flowchart of this research.

From August 2017 to July 2020, 2126 patients with pulmonary diseases who underwent the 7-AABs panel test were identified. During this period, 123 patients were lost to follow-up, 16 died of indefinite causes, 143 patients suffered other malignancies, 13 patients had previously undergone antitumor therapy, and 890 patients are still under observation; these patients were excluded from this study. Finally, 933 patients were included with pathological diagnosis. A total of 571 were identified as having lung cancer, including 502 NSCLC and 69 SCLC determined by surgery and/or biopsy, 362 were considered to have benign lung diseases including benign lung nodules (both pathological and clinical where patients whose pulmonary nodules decreased, disappeared, and calcified during the period of follow-up, and patients with solid nodules that remained stable for at least 2 years), tuberculosis, and pneumonia (**Figure 1**).

Approval to use blood samples was obtained from an Institutional Review Board. The project was approved by the Ethics Committee of Xijing Hospital, Fourth Military Medical University (20130121-6).

### Autoantibody Detection

Serum from 5 mL of blood was separated by centrifugation at 4°C and stored in a sterile tube at -80°C (within 4 h of blood sample collection). The 7-AABs panel assay was conducted in our own laboratory (Respiratory Medicine Laboratory, Xijing Hospital). All blood samples were tested simultaneously. All laboratory testers were blinded to the baseline features of the blood samples. Every set of nine patients’ serum was tested within 3 days of collection. A commercially available enzyme-linked immunosorbent assay (ELISA) kit was used according to the manufacturer’s recommendations. The samples and kit components were equilibrated to laboratory temperature and diluted according to the instructions. Overall, 50 uL of diluted serum samples and standards were added to the appropriate wells and incubated for 1 h. The plate was washed three times followed by addition of 50 uL of diluted secondary antibodies anti-human IgG HRP. After half an hour of incubation, the plate was washed three times. The substrate was added and the color development reaction was terminated after 15 min with 50 uL stop buffer. The plate was measured for optical density (O.D.) at 450 nm on a Dynex MRX Revelation microplate reader. The O.D. units were converted to calibrated reference units according to the standard curve.

The serum from all samples was collected before any systemic or antineoplastic treatment such as surgery, chemotherapy, immunotherapy, and so on. Samples were judged to be positive if the measured concentration level of one or more of the seven AABs was above the cutoffs recommended by the previous study.

### Image Acquisition and Analysis

For patients with pulmonary nodules, chest CT was performed using spiral CT volumetric scan technology (both thickness and interval of layers were set to 5 mm), based on which images were reconstructed using multi-plane reconstruction (MPR) technology with a thin layer (0.625 mm), volume reconstruction (VR), and maximum density projection (MIP) methods. Two experienced radiologists worked independently to determine whether pulmonary nodules were benign or malignant. Different radiological signs including vessels, spiculation, lobulated, pleural indentation, and vacuole signs were recorded. No patient data were visible to the readers. If patients with pulmonary nodules had a chest CT examination in our hospital before 2017, the CT images were also used to compare the chronological changes of pulmonary nodules.

### Data Analysis and Statistics

The differences of the 7-AABs panel levels were done using non-parametric tests (Mann-Whitney U Test). The proportion samples were presented with a 95% exact confidence interval (CI) for binomial proportions. The Chi-square test was used to determine whether the proportion of positive results was significantly different between malignant and benign lung diseases. For all the statistical analyses, P<0.05 was considered significant and all tests were two-sided. SPSS (version 26.0), Graphpad 8.0, and Python were used for all analyses. The sensitivity and specificity of single or combined AABs were evaluated by receiver operating characteristic curves (ROC), and diagnostic efficacy between the 7-AABs and model was also compared by ROC curves.

### Model Structure

**Figure 2:**
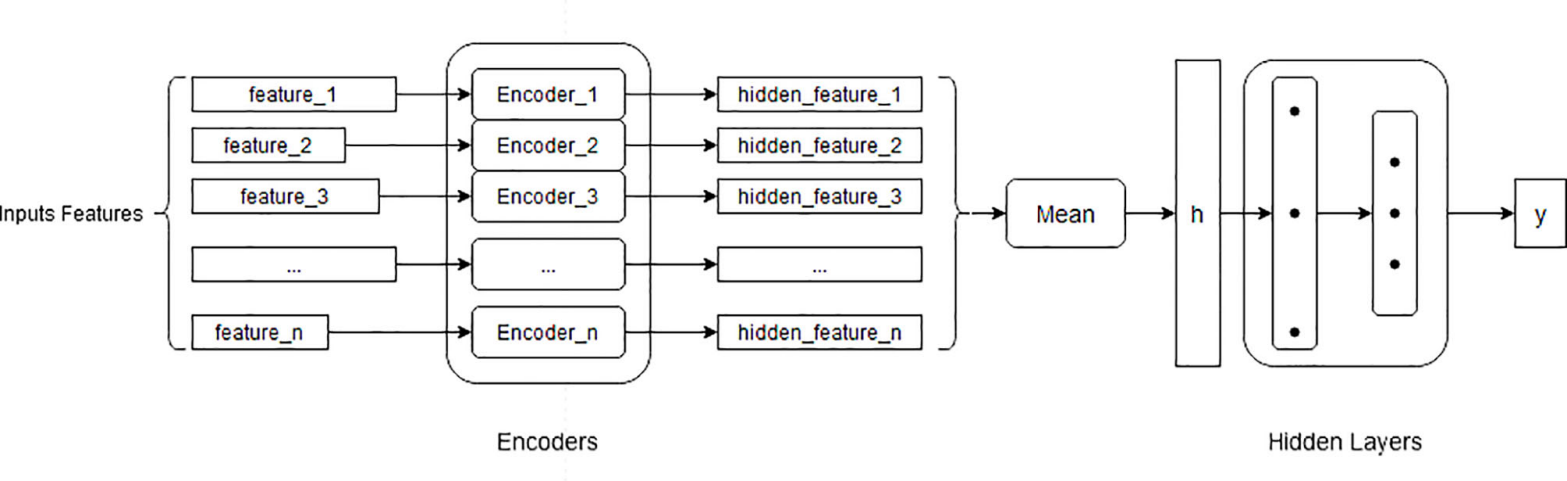
Network analysis construction.

We designed a neural network model combining both continuous and discrete features of clinical patients to predict properties in early lung nodules. The structure of our model is shown in **Figure 2**. The inputs of our model are denoted with *feature_i*, which included clinical features, nodules features, and seven autoantibodies results. The output of the model is the confidence score *y*, a large *y* means high confidence of the positive prediction. Our proposed framework consists of three parts: Encoders, mean pooling, and hidden layers. Encoders are for extracting hidden features of the input features of different types and dimensions, it consists of *n* dense layers (*Encoder_i, i∈1,2,3…,n,n* is the number of input features), each *Encoder_i* mapped one input feature into a hidden feature (*hidden_feature_i, i∈1,2,3…,n*) (Details of the variables are listed in **Table 1**). With the help of the encoders, input features of different types and dimensions are mapped into the same dimension. Mean pooling calculates the average of input vectors, and conveys the result h to the next layers. Hidden layers contain two dense layers, mapping h which integrates all input information of the patient to clinical results *y*. The dense layer is the basic module in our framework, whose parameters includes weight matrix W and bias vector b, taking a linear transformation of the input. It can transform *x* into a vector with the same dimension of bias vector *b*. Formula:


$$Linear(x)=x^{T}W+b$$

**Table 1:** Dimensions and types of variables associated in the model.

Variable	Dimensions	Types
*feature_i*	dim *_i*	Discrete/continuous
*hidden_feature_i*	32	Continuous
*h*	32	Continuous
*y*	1	Continuous

Outputs of each dense layers are activated by the activation function *tanh*.

### Data Process

The concentrations of the seven autoantibodies are continuous variables, hence, we normalized each autoantibody and concatenated seven autoantibodies normalization result scalars into a seven-dimension autoantibodies vector. The formula is as follows *x_i_* refers to the concentration of autoantibodies, *μ* refers to the mean value of any autoantibodies of all patients, and *σ* refers to standard deviation.


$$x_{i}=\frac{{\text{x}}_{\text{i}}-\text{μ}}{\text{σ}}$$

The medical history and characteristics of patients were discrete variables, we transformed them into one-hot vectors.

## Result

### Patients Demographics

To research the efficiency of the 7-AABs panel in lung diseases, we enrolled 933 patients in our study. A total of 571 patients were in the malignant diseases group including 502 with NSCLC and 69 with SCLC. There were 289 women (50.7%) and 282 men (49.3%). A total of 236 (41.3%) of them had a history of smoking. In the benign diseases group, 154 (38.4%) patients were women and 208 (61.6%) were men. Overall, 154 (42.5%) cases had a history of smoking. Benign diseases included pneumonia, tuberculosis, cryptogenic organizing pneumonia, pulmonary fibrosis, pulmonary granulomas, pulmonary sequestration, pulmonary hamartoma, and congenital cystic adenomatoid. Demographics in lung disease are listed in **Table 2**.

**Table 2:** Patient demographics.

	Malignant or borderline diseases (n=571)	Benign pulmonary diseases (n=362)	P valve
Gender, n (%)			0.016
Male	282 (49.4)	208 (57.5)	
Female	289 (50.6)	154 (42.5)	
Age, n (%)			<0.001
≤60	316 (55.3)	258 (71.2)	
>60	255 (44.7)	104 (28.7)	
Smoking history, n (%)			0.715
Ever or current	236 (41.3)	154 (42.5)	
Never	335 (58.7)	208 (57.5)	
Diameter, cm, mean (SD)	16.5 (9.0)	12.4 (5.7)	<0.001
7-AABs			
Positive	347 (60.7)	67 (18.5)	<0.001
Negative	224 (39.3)	295 (81.5)	
Type of malignant lung diseases, n (%)			
NSCLC			
Adenocarcinoma	411 (72.0)		
Squamous carcinoma	91 (15.9)		
SCLC			
Limited stage	65 (11.4)		
Extensive stage	4 (0.7)		
Type of benign lung diseases, n (%)			
Tuberculosis		48 (13.2)	
Pneumonia		123 (34.0)	
Hamartoma		11 (3.0)	
Other benign diseases		180 (49.7)	

### Autoantibody Level in the Malignant Disease Group and Benign Disease Group

The mean concentrations of the AABs including P53, PGP9.5, SOX2, GAGE7, GBU4-5, MAGEA1, and CAGE were 8.87 U/ml, 6.17 U/ml, 7.46 U/ml, 9.59 U/ml, 3.46 U/ml, 6.36 U/ml, and 11.80 U/ml in the malignant group, and 3.25 U/ml, 3.11 U/ml, 3.40 U/ml, 4.08 U/ml, 1.87 U/ml, 3.15 U/ml, and 1.75 U/ml in the benign group, respectively (**Table 3**). The average concentrations levels of all 7-AABs in the malignant diseases group were higher than that in the benign diseases group (*P*<0.001), exhibiting an outstanding performance in the distinction between malignant diseases and benign diseases.

**Table 3:** Concentration and reactivity of 7-AABs in all patients and lung nodules group.

	Full cohort			Patients with lung nodules		
	Patients with malignant diseases (n = 571)	patients with benign diseases (n = 362)	p-value	Malignant lung nodules (n = 459)	Benign lung nodules (n = 285)	p-value
p53 concentration, u/mL, (SD)	8.87 (15.09)	3.25 (5.11)	<0.001	8.208 (14.65)	3.316 (5.128)	<0.001
p53 qualitative diagnosis, n (%)						
Positive	116 (20.3)	18 (5.0)	<0.001	92 (20.0)	9 (3.2)	<0.001
Negative	455 (79.7)	344 (95.0)		357 (80.0)	276 (96.8)	
PGP9.5 concentration, u/mL, (SD)	6.17 (10.67)	3.11 (4.80)	<0.001	6.569 (10.44)	3.025 (3.936)	<0.001
PGP 9.5 qualitative diagnosis, n (%)						
Positive	76 (13.3)	13 (3.6)	<0.001	64 (13.9)	10 (3.5)	<0.001
Negative	495 (86.7)	349 (96.4)		395 (86.1)	275 (96.5)	
SOX2 concentration, u/mL, (SD)	7.46 (12.14)	3.40 (5.92)	<0.001	7.739 (12.67)	3.127 (4.971)	<0.001
SOX2 qualitative diagnosis, n (%)						
Positive	110 (19.3)	21 (5.8)	<0.001	83 (18.1)	14 (4.9)	<0.001
Negative	461 (80.7)	341 (94.2)		376 (81.9)	271 (95.1)	
GACE7 concentration, u/mL, (SD)	9.59 (18.01)	4.08 (7.35)	<0.001	9.34 (17.36)	4.405 (8.020)	<0.001
GACE7 qualitative diagnosis, n (%)						
Positive	98 (17.2)	13 (3.6)	<0.001	81 (17.6)	12 (4.2)	<0.001
Negative	473 (82.8)	349 (96.4)		378 (82.4)	273 (95.8)	
GBU4-5 concentration, u/mL, (SD)	3.46 (5.45)	1.87 (3.57)	<0.001	3.542 (5.693)	1.954 (3.822)	<0.001
GBU4-5 qualitative diagnosis, n (%)						
Positive	97 (16.7)	19 (5.2)	<0.001	78 (17.0)	17 (6.0)	<0.001
Negative	476 (83.3)	343 (94.8)		381 (83.0)	268 (94.0)	
MAGEA1 concentration, u/mL,(SD)	6.36 (12.08)	3.15 (7.19)	<0.001	6.098 (11.44)	3.334 (7.892)	<0.001
MAGEA1 qualitative diagnosis, n (%)						
Positive	76 (13.3)	16 (4.4)	<0.001	58 (12.6)	13 (4.6)	<0.001
Negative	495 (86.7)	346 (95.6)		401 (87.4)	272 (95.4)	
CAGE concentration, u/mL, (SD)	3.44 (8.06)	1.75 (2.54)	<0.001	3.397 (7.891)	1.766 (2.653)	<0.001
CAGE qualitative diagnosis, n (%)						
Positive	53 (9.3)	11 (3.0)	<0.001	42 (9.2)	8(2.8)	<0.001
Negative	518 (90.7)	351 (97.0)		417 (90.8)	277 (97.2)	
Combined test						
Positive, n (%)	347 (60.7)	71 (19.6)	<0.001	274 (59.7)	54 (18.9)	<0.001
Negative, n (%)	224 (39.3)	291 (80.4)		185 (40.3)	231 (81.1)	
AUC	0.7448			0.7476		

### Clinical Value of 7-AABs Panel Assay in Lung Cancer

In this group, the 7-AABs panel showed the highest sensitivity (60.7%, 95%CI 49.9%-68.3%) and specificity (81.5%, 95%CI 75.8%-88.4%). To clarify the effect of tumor parameters, tumor stage and size of nodules or lesions were recorded (**Table 4**). The results showed that the sensitivities of the 7-AABs panel were 61.7% (95%CI 51.9%-69.8%), 58.8% (95%CI 48.2%-67.3%), 71.4% (95%CI 47.8%-88.7%), and 72.7% (95%CI 49.8%-89.3%) in AAH+AIS, stage I and II, stage III, and stage IV, respectively. There was no significantly difference between the subgroup in AAH+AIS and stage I and II (P>0.05). However, the sensitivities in stage III and stage IV were significantly higher than that of patients in stage I and II (P<0.05). Additionally, we conducted the value of the 7-AABs panel in different nodule or lesion sizes which showed that the sensitivities of nodules or lesions whose diameter was ≤8 mm, 9-20 mm, 21-30 mm, and >30 mm were 57.3% (95%CI 24.2%-71.6%), 59.9% (95%CI 43.3%-67.9%), 64.9% (95%CI 44.5%-76.9%), and 80.7% (95%CI 69.1%-89.5%), respectively. There was a significant difference between the >30 mm group and other groups including ≤8 mm, 9-20 mm, and 21-30 mm (*P*<0.05). In this study, we also divided pulmonary nodules into solid nodules, pure ground glass nodules (pGGNs), and mixed GGNs (mGGNs). But we found the sensitivities of the 7-AABs panel were similar among these groups (*P*>0.05).

**Table 4:** Baseline characteristics of the patients with lung nodules.

	Malignant lung nodules (n = 459)	Benign lung nodules (n = 285)	P value
Size of lesions, n (%)			<0.001
φ≤8 mm	83 (18.1)	116 (40.7)	
8 mm <φ≤20 mm	271 (59.0)	133 (46.7)	
20 mm <φ≤30 mm	105 (22.9)	36 (12.6)	
Number of nodules, n (%)			0.103
Single	260 (56.7)	144 (50.5)	
Multiple	199 (43.3)	141 (49.5)	
Composition, n (%)			<0.001
GGO	41 (8.9)	26 (9.1)	
pGGN	103 (22.4)	21 (7.3)	
mGGN	214 (46.6)	15 (5.2)	
Solid	101 (22.0)	223 (78.2)	
Pathologic type, n (%)			
Adenocarcinoma	312 (68.0)		
SCC	39 (8.5)		
AIS or MIA	103 (22.4)		
Neuroendocrine	5 (1.1)		
Lung benign tumor		19 (6.7)	
AAH		141 (49.5)	
Inflammatory lung nodules		72 (25.2)	
Other lung nodules		53 (18.6)	
Stage of lung cancer, n (%)			
0 (AIS)	43 (9.3)		
I	302 (65.8)		
II	51 (11.1)		
III	31 (6.8)		
IV	32 (7.0)		
Imaging features			<0.001
Vessel sign	361 (78.6)	24 (8.4)	
Spiculation sign	179 (39.0)	42 (14.7)	
Lobulated sign	207 (45.1)	51 (17.9)	
Pleural indentation	154 (33.6)	37 (13.0)	
Bubble-like sign	93 (20.2)	26 (9.1)	

### Effectiveness of the 7-AABs Panel in Early Detection of Lung Nodules

**Figure 3:**
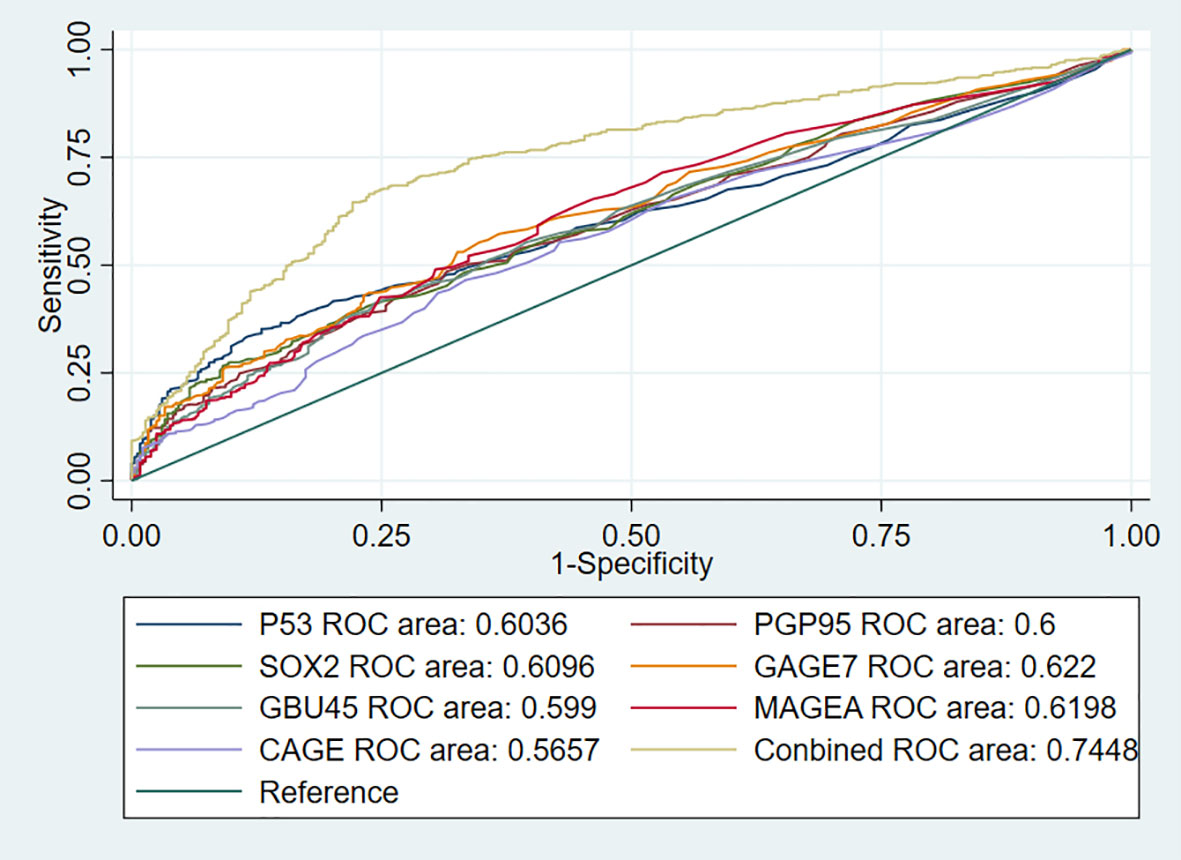
The receiver operating characteristic (ROC) curve analysis of seven autoantibodies and combined test in lung cancer.

**Figure 4:**
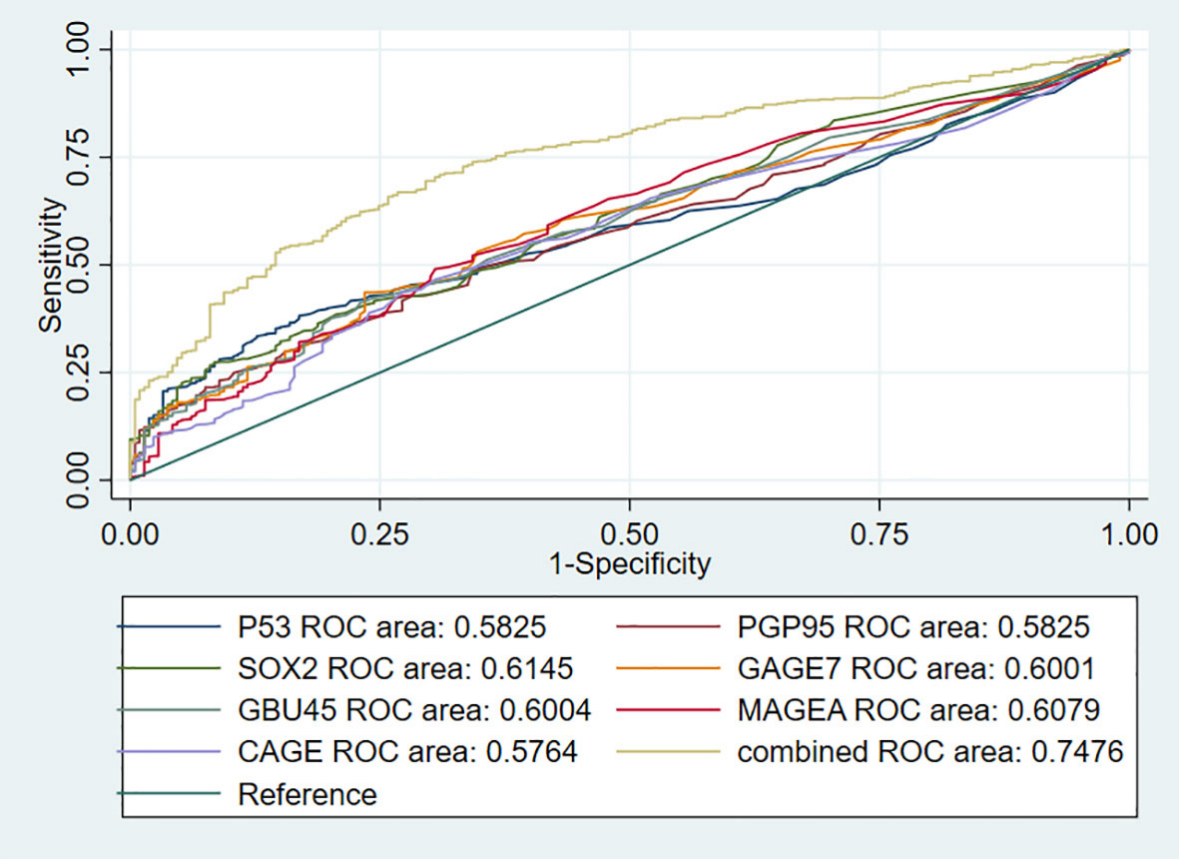
The receiver operating characteristic (ROC) curve analysis of seven autoantibodies and combined test in lung nodules.

To further investigate the performance of the 7-AABs panel in early diagnosis of lung nodules, we identified 744 patients from full cohorts with lung nodules including radiological GGO, GGNs, and (or) solid nodules. Among these patients, 459 (61.6%) were pathologically confirmed with malignancies and 285 (38.4%) were identified as having benign nodules. The sensitivities and specificities of the 7-AABs panel test were 59.7% (95%CI 47.1%-69.4%) and 81.1% (95% CI 65.4%-88.1%) respectively in patients with lung nodules. There was no significant difference between the full cohort and patients with lung nodules. And all seven autoantibodies’ expressions are shown in **Table 3**. The receiver operating characteristic (ROC) curve analysis showed that the 7-AABs exert a great potential on lung cancer diagnosis and lung nodules, and the AUC reached 0.7448 and 0.7476 in all lung diseases and early lung nodules respectively; there is no significant difference in AUC between these two groups (**Figures 3**, **4**).

### Network Model Efficiency in Early Lung Nodules

**Figure 5:**
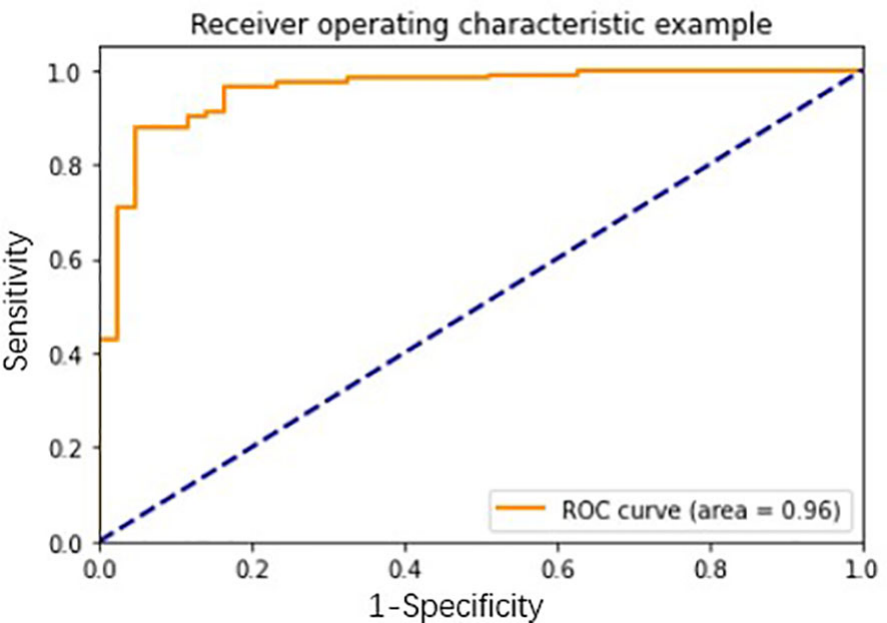
The receiver operating characteristic (ROC) curve analysis of network diagnosis model in lung nodules.

To further utilize clinical and imaging data of lung cancer patients, we built a network diagnosis model based on clinical and imaging information combining 7-AABs data, which could improve both the sensitivity and specificity of lung nodules. We used clinical information (including age, smoking history, cancer history), imaging data (containing size, numbers of lung nodules, property of lung nodules, vessel sign, spiculation sign, lobulated sign, pleural indentation, and bubble-like sign), and 7-AABs panel results to construct the diagnosis model integrally. The model showed an AUC of 0.96 which greatly improved the diagnosis performance (**Figure 5**). The sensitivity, specificity, and accuracy of this model were 0.964, 0.791, and 0.918 respectively, which showed great advantages for patients with lung nodules compared to LDCT and 7-AABs alone. The recall and F1 were 0.83 and 0.86 respectively which showed the good performance and repeatability of this diagnosis model.

## Discussion

As is known, LDCT screening is widely used for the early detection of suspected malignant pulmonary nodules, but it cannot be immediately qualitative. In order to distinguish the malignant from benign nodules, PET/CT, fine needle aspiration biopsy (FNB), transbronchial needle aspiration (TBNA), pathology of sputum, and different types of bronchoendoscope are applied in clinical application. However, these approaches have several problems including high price, inability for early screening, trauma, or poor reliability for microscopic nodules. Certainly, there are some traditional biomarkers such as carcino-embryonic antigen (CEA), neuron-specific enolase (NSE), cytokeratin 19 fragments (CYFRA-21) in lung cancer. The sensitivity of these biomarkers is less than 15% in early detection of lung cancer, which is not feasible for screening of lung cancer (17).

AABs which react with tumor-associated antigens have been discovered in blood samples from various of solid tumors including lung cancer, and have been found in the serum of patients who develop lung cancer at a very early stage. So AABs are potential biomarkers with non-invasive, high sensitivity, and easy-performed properties in early detection of lung cancer (18). In this study, we used a 7-AABs panel (P53, PGP9.5, CAGE, GBU4-5, SOX2, MAGE7, and MAGEA1) to distinguish patients presenting with malignant diseases from ones with benign diseases and further compared the efficiency in the lung nodules group. We found the average concentration levels of the 7-AABs panel in the lung cancer group were significantly higher than that in the benign diseases group, and in the lung nodules group, no distinct results were found. These results are consistent with a previous study (14, 19). It provides evidence that the 7-AABs panel exists dependently in patients with malignant diseases. It suggests that patients whose concentration levels of the 7-AABs were higher in serum could suffer from the higher possibility of malignancy. And the network model combining the 7-AABs could become more efficient to lung cancer patients.

Some AABs panels have been reported in previous literature. In the USA, EarlyCDT-Lung was the first reported AABs panel for detection of lung cancer. In comparison to a 6-AABs panel, a 7-AABs panel had higher sensitivity. Interestingly, the test specificity was improved by changing from a 6-AABs panel to the current 7-AABs panel (20). Now, the 7-AABs panel (EarlyCDT-Lung) is performed in routine clinical application in the USA. Additionally, apart from the EarlyCDT-Lung, there are other AABs panels under assessment in clinical application. A previous study by Yao et al. reported the clinical validation of a new AABs panel including SMOX, NOLC1, MALAT1, and HMMR. It showed that the sensitivity and specificity were 47.5% and 97.3% (21). In order to create a preferable AABs panel, a different AABs panel (P53, PGP9.5, GAGE, GBU4-5, SOX2, MAGE7, and MAGEA1) was applied by a large-scale ELISA screening in lung cancer patients in China. Ren et al. found that the total sensitivity and specificity of the 7-AABs panel were 61% and 90% in lung cancer. And the sensitivities of the 7-AABs panel were 62% and 59% in stage I and stage II lung cancer patients with pulmonary nodules. In this study, we also tested the reliability of the 7-AABs panel in lung cancer, and found a sensitivity of 60.7% in the lung cancer group with a specificity of 81.5%. Meanwhile, we confirmed the clinical value of the 7-AABs panel in early detection of lung nodules. The sensitivity and specificity of the 7-AABs panel were about 59.7% and 81.1% in patients with pulmonary nodules, which is consistent with the majority of previous studies about the 7-AABs panel and the results of the EarlyCDT-Lung assay (22, 23). But the specificity in our study was lower than Ren’s study. This is probably due to various factors. First, the different results are often restricted by the amounts of samples. Second, the patients enrolled from different regions may have different characteristics in the tumorigenesis process. Maybe the clinical manifestation and biochemical characteristics of nodules for populations are different. Third, the composition ratio of pathology, morphology, and size of nodules is different, e.g., there are more AAH and AIS in our study. These may be the potential reasons for the difference.

In the current study, there was no statistical significance about the positive rate of the 7-AABs panel in patients presenting with nodules with a diameter of <8 mm, 9-20 mm, and 21-30 mm. It indicates that the 7-AABs panel served as a promising method of judging the nature of the <8 mm nodules. Furthermore, it confirms that the 7-ABBs panel is valid and can be utilized for lung screening. Some previous research reported that the positive rate of the 7-ABBs panel did not correlate with stage (24). But our study obtained opposite results. This study found that the sensitivities of lung cancer patients with stage III and stage IV were significantly higher than in patients with stage I and II. It probably meant that advanced lung cancer would release more tumor-related antigens, then more AABs would be produced. Then we can assume that the sensitivity in patients with advanced lung cancer would be higher than that in patients in the early stage. The results of morphology subgroup analyses confirmed the assumption. As we know, the pathological types of GGNs are mostly pre-invasion lesions including atypical adenocarcinoma hyperplasia (AAH), adenocarcinoma *in situ* (AIS), and minimally invasive adenocarcinoma (MIA). But there are more invasive adenocarcinomas(IACs) in malignant solid nodules. This study showed that the ratios of IAC were 9.0% (67/744), 16.7% (124/744), 30.8% (229/744), and 43.4% (323/744) in GGO pGGNs, mGGNs, and solid nodules, respectively. And it illustrated that the sensitivities ranged from 52.1% to 72.3% in patients, with a sensitivity of 52.1% in the GGO group, 61.3% in the pGGNs group, 72.3% in the mGGNs group, and 63.2% in the solid group. So it further confirmed the assumption that the sensitivity of the 7-AABs panel would improve with more invasive lesions. However, more studies are needed to confirm the assumption. In our study, we also found that the sensitivity of patients with a lesion diameter of >3 cm was significantly higher than that in patients with nodules of <3 cm. The results meant that the 7-AABs panel could not only be applied in early detection of patients with modules but also in diagnosis of advanced lung cancer.

Although the 7-ABBs panel has been approved by China Food and Drug Administration, it lacks large-scale clinical studies to further choose the optimum subgroup population. Except for the 7-AABs panel, other liquid biomarkers including circulating tumor cells (25, 26), circulating-tumor DNA (27), microRNAs (28), and DNA methylation (29) are gradually emerging. Status also showed promising results for the non-invasive detection and diagnosis of lung cancer. Finding the optimal combination with other information of patients to facilitate the early detection of lung cancer is imperative.

Machine learning is emerging as the best method for large amounts of samples and data (30). Network learning is a type of machine learning that can process different types of data at the same time, including continuous variables and discrete variables, so it has its own unique advantages for the construction of diagnostic models. To further utilize the results of the 7-AABs, given their high specificity and the significant roles of lung nodules in early lung cancer, we wanted to build a comprehensive model to enhance the comparatively low sensitivity of 7-AABs alone, hence we constructed a diagnostic model of early pulmonary nodules with high diagnostic efficiency by analyzing the imaging characteristics and clinical characteristics of lung cancer patients and combining it with the advantages of the seven serum antibodies of patients. And compared with previous models like the Mayo model and Brock model (31, 32), we took advantage of almost all the patients` information that we could get plus high specificity 7-AABs results, rather than just several characteristics of nodules. Therefore, we found great performance in comparison with previous models in the fields of sensitivity, specificity, and accuracy. However, there are several aspects that we need to improve on in our study. First, the samples in our research still need to be collected in a larger scale to verify our results. Second, we are considering adding more test results such as CEA, CTCs, and DNA methylation level to maintain our model, and to further increase the sensitivity and specificity.

In summary, this study confirmed the clinical value of the 7-AABs panel in early detection and diagnosis of lung cancer. When combined with clinical and imaging data, the model could significantly improve sensitivity and reduce the FPR of the 7-AABs panel or LDCT screening alone. Meanwhile, we first found the correlation between stage and sensitivity of the 7-AABs panel. Maybe the 7-AABs panel can serve as an adjunctive non-invasive biomarker test capable of distinguishing malignant from benign nodules.

## Data Availability Statement

The original contributions presented in the study are included in the article/supplementary material. Further inquiries can be directed to the corresponding author.

## Ethics Statement

The studies involving human participants were reviewed and approved by Ethics Committee of Xijing Hospital, Fourth Military Medical University. The patients/participants provided their written informed consent to participate in this study.

## Author Contributions

JZ designed this study. NC and TY collected the data. LX and YL analyzed patients’ clinical information. HX and WZ analyzed imaging features. YZha, YC, and QS analyzed and interpreted the data. SQ wrote the manuscript. YZho, JY, and XY revise the manuscript. All authors reviewed the manuscript. All authors contributed to the article and approved the submitted version.

## Funding

This work was supported by the Shaanxi Research and Development Plan (2017ZDXM-SF-044), China.

## Conflict of Interest

QS is employed by Amoy Diagnostics Co. Ltd.

The remaining authors declare that the research was conducted in the absence of any commercial or financial relationships that could be construed as a potential conflict of interest.

## Publisher’s Note

All claims expressed in this article are solely those of the authors and do not necessarily represent those of their affiliated organizations, or those of the publisher, the editors and the reviewers. Any product that may be evaluated in this article, or claim that may be made by its manufacturer, is not guaranteed or endorsed by the publisher.
